# Income-Nutrition Gradient and Intrahousehold Allocation in Rural Pakistan

**DOI:** 10.1007/s10995-023-03633-4

**Published:** 2023-03-29

**Authors:** Haseeb Ahmed, Hina Khalid

**Affiliations:** 1 Inclusive Rural Transformation and Gender Equality Division, Food and Agriculture Organization of the United Nations (FAO), Viale delle Terme di Caracalla, 00153 Rome, Italy; 2 Washington State Department of Social and Health Services, Research and Data Analysis Division, Olympia, United States

**Keywords:** Income-nutrition gradient, Son-preference, Child health, Intra-household allocation, Pakistan, South Asia

## Abstract

**Objective:**

To estimate the relationship between household income and child health outcomes for male and female children, aged 0–5 years, in rural Pakistan.

**Method:**

The study uses 2014 round of Pakistan Rural Household Panel Survey (PRHPS) and regression analyses to estimate the relationship between household income and child health outcomes for male and female children in rural Pakistan.

**Results and Policy Implications:**

We find that increase in income is associated with an increase in child weight-for-age and weight-for-height z-scores, and reduction in the likelihood of a child being underweight or wasted. However, our results suggest that these gains associated with an increase in income are greater for male children as compared to female children. These differences in income-nutrition gradient can be explained by the gender-differences in consumption of health inputs (e.g., food intake, vaccinations, and nutritional supplements) associated with an increase in income. Our results indicate the need for policy instruments that can encourage an equitable resource allocation within households.

## Significance

This study documents the gender bias in income-health relationship for children in rural Pakistan. Our results imply that interventions that target poverty alleviation at the household level may not have equitable impacts on all members of the household because of possible ‘son-preference’. This suggests that there is a need to design gender-sensitive interventions to ensure that improvements in nutritional outcomes are shared across genders within households.

## Introduction

Undernutrition poses a major development challenge for low- and middle-income countries (LMICs). It has been linked to high mortality rates among children; approximately half of the deaths of children under the age of 5 years can be attributed to under nutrition (Black et al., 2008; UNICEF, 2019). Poor nutrition starts in-utero and can result in low birth weight and an increased susceptibility to communicable and non-communicable diseases (Pena & Bacallao, 2002). Children who are undernourished are often stunted, wasted, or underweight. Low weight-for-height (wasting) signals acute growth disturbance, low height-for-age (stunting) reflects long term growth faltering, and low weight-for-age (underweight) reflects a combination of short- and long-term effects (Marcoux, 2002). Such children are also more likely to experience poor cognitive development and reduced productivity in adulthood (WHO, 2020).

The relationship between income and child health, and the mechanisms through which income and child health are related are an important policy-related issue that has been the focus of a large empirical literature (e.g., Behrman & Deolalikar 1987; Burgess et al., 2004; Case et al., 2002; Currie & Stabile, 2003; Currie et al., 2007; Frijters et al., 2005; Smith & Haddad, 2002). Furthermore, literature has documented several dimensions of inequitable intrahousehold resource allocation between male and female children, especially in the context of South Asia, (Abdullah & Wheeler, 1985; Behrman, 1988a, 1988b; Cowan & Dhanoa, 1983; Miller 1997; Gupta, 2016; Javed & Mughal, 2019; Jayachandran & Pande, 2017). However, little attention has been given to understanding differences in the income-nutrition gradient across male and female children, especially in a developing country context. In this paper, using Pakistan as a case, we investigate the relationship between income and child health outcomes and if higher income translates into increased consumption of an array of health inputs for both male and female children.

Income is often linked to poor health outcomes, with studies suggesting ‘wealthier is healthier’ (Smith & Haddad, 2002). Social scientists often point to the “*gradient* in health status”, which refers to “the phenomenon that relatively wealthier people have better health and longevity” and argue that this gradient is “evident throughout the income distribution” (Case et al., 2002). For instance, using data on children from the US National Health Interview Survey, Case et al. (2002) provides evidence of a significant positive income gradient, where children in poorer families have worse health outcomes as compared to those from richer families with this relationship becoming more pronounced as children grow older (Case et al., 2002). Similarly, in another study focusing on Canadian children, Currie and Stabile (2003) find that the income of a family is negatively related to being in poor health and the income gradient increases with child age. This income-nutrition gradient holds when an increase in income is accompanied by an increase in consumption of an array of health inputs including better caloric intake and dietary diversity (Agrawal et al, 2019; Black et al., 2008; Mahmudiono et al., 2017; Melaku et al., 2018; Moges et al., 2015; Nnyepi, 2007; Rah et al., 2010; Vella et al., 1994).

Yet, other studies find that income does not lead to improved health outcomes*.* For instance in studies focusing on British children, Burgess et al (2004) and West (1997) find that the gradient between family income and child health is very small and the slope of the gradient does not increase with the age of the child (Burgess et al., 2004; West, 1997). Similarly, using a sample of 13,000 children in England, Currie et al (2007) find that while there is a family income gradient in child health, the size of the gradient is very small. Other studies drawing on panel data across countries, also do not find strong evidence that supports the link between income and health outcomes (Adams et al., 2003; Frijters et al., 2005; Meer et al., 2003).

The contribution of this paper to this above-mentioned literature is twofold. First, this paper contributes to the literature by estimating the income-nutrition gradient for male and female children, aged 0–5 years, in rural Pakistan. While the literature has documented instances of intrahousehold gender inequality in South Asia in related dimensions, such as height advantage for first-born sons (Behrman, 1988a; Jayachandran & Pande, 2017), participation in household decision-making (Javed & Mughal, 2019), and voting (Cheema et al., 2022), the differences in income-nutrition gradient across genders within households are not documented. Second, the paper estimates the relationship between income and health inputs for both male and female children to understand the mechanisms that may underlie the gradient and the gender differences in the gradient.

We find that while increases in income are associated with betterment in several child health outcomes, the gains are consistently greater for male children as compared to female children. These differences in income-nutrition gradient between boys and girls can be explained through inequality in intra-household resource allocation. Indeed, we find that gains associated with increased income in terms of intake of individual food items, vaccines and vitamin A supplements are greater for boys as compared to the girls. This finding with respect to income-nutrition differences among male and female children is especially interesting, as currently little work as has been done to document these gendered income-nutrition patterns.

## Data and Methods

This paper uses the data from the 2014 round of Pakistan Rural Household Panel Survey (PRHPS). PRHPS is a panel survey from 2012 to 2014 that collected information from 1876 rural households in three provinces (Sindh, Punjab, and Khyber Pakhtunkhwa) of Pakistan (IFPRI, 2017). However, information on child nutritional outcomes and food intake are collected for the 2014 round specifically. Therefore, we use the 2014 wave of the PRHPS data for our analysis.

**Fig. 1:**
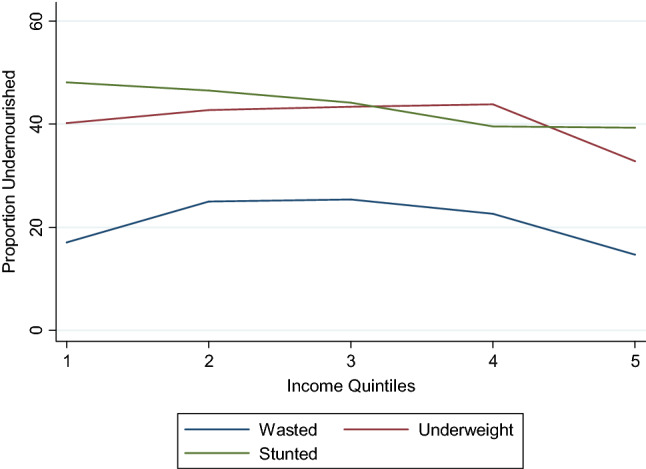
Proportion of child nutritional outcomes (stunted, underweight and wasted) and income quintiles

Table 1 describes the data and Table 2 provides summary statistics. Height and weight of about 900 children below or equal to the age of 5 years was measured by the survey team. Height-for-age (*HAZ*), weight-for-age (*WAZ*) and weight-for-height (*WHZ*) z-scores were calculated from these height and weight measurements based on the World Health Organization’s child growth standards (WHO, 2006). Outcome variables, *Stunted*, *Underweight* and *Wasted*, are dummy variables equal to 1 if HAZ, WAZ and WHZ are less than − 2, zero otherwise (using the WHO, 2006). In our sample, 43%, 41% and 21% of the children are *Stunted*, *Underweight* and *Wasted*, respectively. Incidence of *Stunted*, *Underweight* and *Wasted* children is at 48%, 43% and 21% for boys and 38%, 38% and 20% for girls, respectively. Pairwise t-tests suggest that means of *Stunted* and *Underweight* are statistically different (at 1% level of significance) between male and female children, with burden of undernutrition more on male children in rural Pakistan. Means of *Wasted* are statistically indifferent. We plot the incidence of the three anthropometric outcomes against quintiles of household income (Fig. 1). From Fig. 1, the relationship between income and child anthropometric outcomes is not entirely clear.

**Table 1:** Data description

Variable	Description
*HAZ*	Height-for-age z-scores calculated based on 2006 World Health Organization’s child growth standards
*WAZ*	Weight-for-age z-scores calculated based on 2006 World Health Organization’s child growth standards
*WHZ*	Weight-for-Height z-scores calculated based on 2006 World Health Organization’s child growth standards
*Stunted*	Indicator variable = 1 if HAZ < − 2, 0 otherwise
*Underweight*	Indicator variable = 1 if WAZ < − 2, 0 otherwise
*Wasted*	Indicator variable = 1 if WHZ < − 2, 0 otherwise
*Per Capita Income*	Log of household’s per capita consumption expenditure (including expenditures on food, electricity, communication, transport, health, schooling, clothes other, etc.) aggregated at the annual level, calculated as ln(*x*)
*Child Food Intake*	Count of the number of different types of foods (milk, meat, eggs, cereal, wheat, pulses) consumed by the children (of age less than 5 years)
*Meat Intake*	Indicator variable = 1 if child ate meat (poultry, beef, mutton, lamb, or fish) in the last 24 h, 0 otherwise
*Eggs Intake*	Indicator variable = 1 if child ate an egg in the last 24 h, 0 otherwise
*Milk Product Intake*	Indicator variable = 1 if child ate any milk product (yogurt, butter, cream etc.) in the last 24 h, 0 otherwise
*Pulses intake*	Indicator variable = 1 if child ate pulses in the last 24 h, 0 otherwise
*Cereal Intake*	Indicator variable = 1 if child ate any cereals (wheat, rice, maize, semolina, etc.) in the last 24 h, 0 otherwise
*Vaccines*	Count of the number of vaccines a child received
*Vitamin A intake*	Indicator variable = 1 if child took vitamin A supplement in last two weeks, 0 otherwise
*Household size*	Number of adult and child members of the household
*Mother’s Education Levels*	Separate indicator variables for mother’s primary and secondary education achievement
*Father’s Education levels*	Separate indicator variables for father’s primary, secondary and tertiary education achievement
*Age Household Head*	Age of the household head
*Child’s Gender*	Indicator variable = 1 if child under five years of age is a boy, 0 if a girl
*Child’s Age*	Age of the child in months

**Table 2:** Summary statistics

	Mean	SD
*HAZ*	− 1.68	1.72
*WAZ*	− 1.68	1.62
*WHZ*	− 0.77	1.80
*Stunted*	0.43	–
*Underweight*	0.41	–
*Wasted*	0.21	–
*Child Food Intake*	1.16	1.11
*Meat Intake*	0.07	–
*Milk Products Intake*	0.52	–
*Eggs Intake*	0.14	–
*Cereal Intake*	0.30	–
*Pulses Intake*	0.09	–
*Vaccines*	8.87	4.74
*Vitamin A Intake*	0.15	–
*Per capita Income (Rs.)*	20,986	18,263
*Household Size*	9.01	4.48
*Mother’s Education (Primary)*	0.65	–
*Mother’s Education (Secondary)*	0.20	–
*Father’s Education (Primary)*	0.24	–
*Father’s Education (Secondary)*	0.21	–
*Father’s Education (Tertiary)*	0.03	–
*Age Household head (years)*	44.90	14.49
*Child’s Gender (Boy* = *1)*	0.52	–
*Child’s age (months)*	24.5	17.2

Annual household income is calculated as household’s per capita consumption expenditure (including expenditures on food, electricity, communication, transport, health, schooling, clothes other, etc.) aggregated at the annual level. This annual consumption expenditure, which we refer to as income in rest of the manuscript, is divided by the number of household members to obtain per capita yearly income of the household. In our sample, the mean per capita income is Rs. 20,986 (about $210 according to PKR-USD exchange rate in 2014). Therefore, an average household in our sample earns less than $2 a day. Other important variables included in the analysis are *mother’s education levels*, *father’s education levels*, *child’s age* and *gender*, *age of the household head* and *region of residence*.

The survey also includes information on the number of food items a child (of ≤ 5 years of age) consumed in the last 24 h. This information was collected from the mothers of the children. We use this information to develop the variable, *Child Food Intake*, which is a Guttman scale of number of food items (including cereals, meat, milk, eggs, and pulses). This variable is used to reflect dietary quality/diversity of children in the household. Mean of *Child Food Intake* is 1.16 items with 0 being the minimum and 5 being the maximum.^1^ Means for individual items are also presented in Table 2. 7% of the children consumed meat (poultry, fish, beef, mutton or lamb) in the last 24 h. This average consumption is 52% of for milk products (ghee, butter, yogurt), 9% for pulses, 30% for cereals (rice, wheat, maize, semolina etc.) and 14% for eggs.

Consumption of *Vaccines* and *Vitamin A Supplements* could be further mechanisms (health inputs) that may affect income-nutrition relationship and are included in the analysis. Children are given an average of 8.89 vaccinations (including OPV, Pentavalent vaccines, Pneumococcal vaccines, BCG vaccine, and measles vaccinations) with a minimum of 5 and maximum of 13. 15% of the children received a *Vitamin A Supplement* in the last two weeks of the survey.

For empirical analysis, we use several econometric models to understand income-nutrition gradient in rural Pakistan. First, we use OLS regressions to estimate the relationship between income and nutritional outcomes such as HAZ, WAZ, and WHZ. Then, we use Probit regressions to investigate if children in poorer households are more likely to be stunted, underweight and wasted. Furthermore, we use Probit and Negative Binomial regressions to estimate the impact on health inputs, to investigate which of the mechanisms potentially explain the underlying income-nutrition gradient.

Following Brown et al. (2019) and Currie et al. (2007), we introduce other sources of heterogeneity in terms of household-level and individual-level characteristics that can be expected to enhance power for predicting child health outcomes. We run several econometric regressions of the following form:

1 $${y}_{ij}=f(Incom{e}_{j}, {X}_{j},{Z}_{ij})+{e}_{ij}$$

where ${y}_{ij}$ is (a) child anthropometric outcomes of the child *i* in household *j* or (b) health inputs (food intake, vaccinations, supplements) consumed by children (aged ≤ 5 years) of household *j*. Functional form of the regression, $f\left(.\right)$, depends on structure of the outcome variable. For example, for child anthropometric outcomes that are standardized indices, linear regressions are used to estimate the relationship between child health and income. However, Poisson and Probit models are used when outcome variables are count or indicator variables, respectively.

$Incom{e}_{j}$ is natural log of yearly per capita household expenditure. ${X}_{j}$ control for household-level variables, e.g., age and gender of household head and district of residence and ${Z}_{ij}$ control for individual-level variables like child’s age and gender and education levels of child’s father and mother. The district of residence fixed effects control for unobservable district-level characteristics (e.g., the unobserved quality of child or maternal health services in a district) that could be correlated with incomes as well as outcome variables.

These regressions are estimated for the full sample, as well as separately for boys and girls within the households. Wald tests are conducted to test the significance of differences in the income-nutrition gradient across boys and girls within households. Standard errors are clustered at the household-level to control for intra-household correlation in errors.

## Results

Table 3 shows the results for the linear regression with *HAZ*, *WAZ*, and *WHZ* as dependent variables. In Column 1, Table 3, we provide results for the full sample. We find no significant relationship between *HAZ* and *Per Capita Income* of households. In column 2 and 3 of Table 3, we provide results disaggregated by gender of the children. Again, for *HAZ*, we do not find a statistically significant positive relationship between income and *HAZ* in our disaggregated regressions.

**Table 3:** Income-nutrition gradient

	Full Sample (1)	Boys (2)	Girls (3)	${\chi }^{2}$ Statistic (P-value)
Height-for-age regressions				
*Per Capita Income*	− 0.047 (0.145)	0.140 (0.175)	− 0.299 (0.202)	1.06 (0.30)
No. of Obs	856	445	411	
Weight-for-Age regressions				
*Per Capita Income*	0.357*** (0.115)	0.470** (0.182)	0.171 (0.143)	10.04*** (0.006)
No. of Obs	857	443	414	
Weight-for-Height regressions				
*Per Capita Income*	0.403** (0.161)	0.427** (0.211)	0.266 (0.293)	7.96** (0.018)
No. of Obs	799	417	382	

Standard errors are clustered at the household level

Control variables include *Household Size, Age of Household Head, Child’s Gender* (in column 1 only), *Child’s Age in Months* (in column 1, 2 and 3), *Mother’s Education, Father’s Education and District dummies*

Column (4) provides test statistics for Wald tests that test the statistical significance of coefficients associated with boys vs girls’ regressions in column (2) and (3)

***, **, *Indicate significance at 1, 5 and 10% respectively

For the Weight-for-Age regressions, we find that a log-point increase in *Per Capita Income* is associated with an increase in *WAZ* scores of 0.357, statistically significant at 1% level of significance (p-value < 0.01). This coefficient is 0.470 for boys, statistically significant at 5% (p-value < 0.05), and 0.171 for girls, not statistically significant at conventional levels (Table 3, columns 2 and 3). We observe a similar pattern of relationship between income and *WHZ* scores. One log-point increase in per capita income is associated with increase in *WHZ* score of 0.40, statistically significant at 5% level of significance (p-value < 0.05). This coefficient is 0.427 for boys, statistically significant at 5% (p-value < 0.05) level of significance, and 0.266 for girls, not statistically significant at conventional levels (Table 3, columns 2 and 3).^2^ This difference in magnitude of income-nutrition gradient among boys and girls suggests that the gains from income in terms of nutrition may not be equally dispersed between different members of the households and point towards inequitable intra-household allocation of resources.

Table 4 provides the elasticities for the relationship between income and incidence of *Stunted*, *Underweight* and *Wasted*. Again, consistent with the results in Table 3, we find no significant relationship between income and stunting outcome. However, for *Underweight* outcome, we find that a 10% increase in *Per Capita Income* is associated with a reduction in the likelihood of a child being underweight by 1.8% (statistically significant at 10% level of significance). This elasticity is − 0.161 and − 0.091 for boys and girls (not statistically significant at conventional levels), respectively. Furthermore, we find that a 10% increase in *Per Capita Income* is associated with a 4.4% reduction in the likelihood of being *Wasted*. This elasticity is − 0.416 for boys (statistically significant at 10% level of significance) and − 0.342 for girls (not significant at conventional levels. Again, we observe that the magnitude of coefficients for boys are greater than that of girls, but the magnitude of difference is less than what we observed in Table 3.

**Table 4:** Relationship between income and child nutrition outcomes—elasticities from probit regressions

	Full Sample (1)	Boys (2)	Girls (3)	${\chi }^{2}$ Statistic (P-value)
Stunting				
*Per Capita Income*	0.017 (0.097)	− 0.076 (0.145)	0.234 (0.162)	3.25 (0.196)
Number of Obs	856	445	411	
Underweight				
*Per Capita Income*	− 0.186* (0.098)	− 0.164 (0.131)	− 0.091 (0.163)	2.01 (0.366)
Number of Obs	857	443	414	
Wasted				
*Per Capita Income*	− 0.44*** (0.15)	− 0.416* (0.218)	− 0.342 (0.245)	5.17* (0.075)
Number of Obs	799	417	382	

Standard errors for elasticities are estimated using the delta method

Control variables include *Household Size, Age of Household Head*, *Mother’s Education, Father’s Education, Child’s gender* (in column 1 only)*, Child’s age* (in column 1,2 and 3 only),* and District dummies*

Column (4) provides test statistics for Wald tests that test the statistical significance of coefficients associated with boys vs girls’ regressions in column (2) and (3)

***, **, *Indicate significance at 1, 5 and 10% respectively

To understand the mechanisms behind the gradient between income and nutrition, we investigate the potential of an increase in income in improving dietary intake of children, vaccination adoption and vitamin A supplement use. Indeed, these variables may partially capture some of the many channels through which income affects child health and nutrition. Table 5, Row 1 shows that a 10% increase in *Per Capita Income* is associated with 1.7% (statistically significant at 5% level of significance) increase in the likelihood of consumption of additional food items. When disaggregated with respect to gender, we find that elasticities are 0.235 (statistically significant at 5% level of significance) and 0.149 (not statistically significant at conventional levels) for boys and girls, respectively.

**Table 5:** Relationship between Income and health inputs—elasticities

	Full Sample (1)	Boys (2)	Girls (3)	${\chi }^{2}$ Statistic (P-value)
Count of food intake regressions				
*Per Capita Income*	0.177** (0.077)	0.235** (0.119)	0.148 (0.097)	14.87*** (0.000)
Eggs intake (Yes/No)				
*Per Capita Income*	0.811*** (0.292)	1.16** (0.456)	0.414 (0.325)	20.66*** (0.000)
Meat intake (Yes/No)				
*Per Capita Income*	1.15*** (0.396)	1.053* (0.640)	1.31** (0.534)	2.35 (0.308)
Milk product intake (Yes/No)				
*Per Capita Income*	0.139 (0.111)	0.299 (0.193)	0.084 (0.191)	1.68 (0.432)
Cereal intake (Yes/No)				
*Per Capita Income*	0.027 (0.139)	0.480* (0.254)	− 0.495 (0.296)	8.91** (0.011)
Pulses intake (Yes/No)				
*Per Capita Income*	0.162 (0.151)	− 0.116 (0.212)	0.395* (0.222)	3.77 (0.153)
Count of vaccines				
*Per Capita Income*	0.090* (0.046)	0.156* (0.082)	0.044 (0.053)	5.91* (0.079)
Vitamin A supplements				
*Per Capita Income*	0.139 (0.160)	0.166 (0.190)	− 0.059 (0.239)	2.53 (0.282)

N = 856 for full sample (445 boys and 411 girls)

***, **, *Indicate significance at 1, 5 and 10% respectively

Standard errors for elasticities are estimated using the delta method

Elasticities for *Count of Food Intake* and *Number of Vaccines* regressions are based on Negative Binomial regressions

Elasticities for all other regressions are based on Probit models

Control variables include *Household Size, Age of Household Head*, *Mother’s Education, Father’s Education, Child’s gender* (in column 1 only)*, Child’s age*,* and District dummies*

Column (4) provides test statistics for Wald tests that test the statistical significance of coefficients associated with boys vs girls’ regressions in column (2) and (3)

Similarly, we also estimate the relationship between income and individual dietary components like meat, milk products, cereals, eggs, and pulses (Table 5). We find that a 10% increase in *Per Capita Income* is associated with 8.1% increase in the likelihood of an egg being consumed in the last 24 h. Again, the coefficient of this relationship for boys is almost three times that of the girls. Similarly, coefficients for milk products and cereals are larger for boys, though not statistically significant. We also find that a 10% increase in income is associated with an 11.5% increase in the likelihood of consuming meat in the last 24 h. This increase is fairly equally distributed among boys and girls in our sample.

Table 5 also provides the results for the relationship between *Per Capita Income* and *Vaccines* and *Vitamin A Supplements.* We find that a doubling of per capita income is associated with an increase of 9% in the likelihood of a child given additional vaccine, statistically significant at 10% level of significance. The elasticity for boys, 0.156 (statistically significant at 10% level of significance), is four times greater than that of the girls, which is 0.044 and not statistically significant at conventional levels. Regressions for *Vitamin A Supplements* do not appear to be significantly related to increase in per capita income. In most cases, Table 5 results show that the gradient for boys is greater than that of girls and is suggestive of inequality in intra-household resource allocation. Furthermore, these results help explain the differences in magnitudes of income-child health relationship among boys and girls in rural Pakistan.

## Discussion

In this paper, using Pakistan as a case, we investigated the relationship between income and child health outcomes and if higher income translates into more diversified nutritional intake among children. Due to a son-preference phenomenon in South Asia, we disaggregated these results by gender to understand if gains from increases in income are equally distributed between different members of the household. We find that an increase in *per-capita income* is associated with an increase in *WAZ* and *WHZ* scores but not *HAZ* scores and the magnitude of income-nutrition gradient among boys is higher. In terms of the diversity of nutritional intake, we find that an increase in income is associated with improved dietary diversity and increased likelihood of consumption of most foods. Again, gains appear to be higher among male children as compared to female children.

Previous research provides mixed evidence for the presence of an income-nutrition gradient, with studies on both sides of the spectrum. Our findings are in line with the literature that suggests that income is related to improved nutritional outcomes among children (Case et al., 2002; Currie & Stabile, 2003; Smith & Haddad, 2002). However, we did not find significant results for the HAZ score. We suspect these results may be due to the cross-sectional nature of our data which does not allow us to study long term changes, which are more characteristic of child stunting (McGovern et al., 2017). Additionally, research in the context of South Asia highlights the presence of a ‘son preference’, where male children are preferred over daughters. Yet, little attention had been given to investigating the presence of son-preference in income-nutrition relationship, especially in the context of Pakistan. Our findings support the existing literature on son-preference (Abdullah & Wheeler, 1985; Brown et al., 1982; Carloni, 1981; Chen et al., 1981; Javed & Mughal, 2019; Jayachandran & Pande, 2017) and add to it by examining the sex disparities in income-nutrition relationships for Pakistan. This finding with respect to sex disparities in the income-nutrition relationship is unique, as little work has been done to document the gendered differences in income-nutrition gradient, especially in Pakistan.

There are several implications of our findings. First, an emphasis on effective nutritional programming can help in improving health outcomes. Nutritional programming focuses on the relationship between critical windows in the development of early life and the nutritional environment at that time. Specifically, undernourishment in the womb can be linked to severe developmental challenges later in life. The fetus makes adjustments based on the predicted environment available after birth, and the prenatal nutritional environment is the main source of information for making these predictions (“Nutritional programming during pregnancy and in early life”, 2011). Work in Gambia finds that for populations with high rates of under-nutrition in early life, there is greater susceptibility to infection related mortality and morbidity later in life (Moore, 2017). According to the National Nutrition Survey (NNS) 2018 for Pakistan (Government of Pakistan & UNICEF, 2019), women of reproductive age in Pakistan have severe nutritional deficiencies. Approximately 14.4% were underweight and 13.8% were obese. The prevalence of anemia very high (41.7%) and Vitamin A deficiency estimated around 22.4%. (UNICEF, 2022) For Pakistan, nutritional programming with an emphasis on gender can go a long way. Our findings also point towards a gender bias in income-health relationship, as well as consumption of health inputs like dietary diversity, suggesting a need to incorporate gender in nutritional programming. In recent years, there have been a number of interventions in Pakistan to improve child anthropometrics. Some of these have included school health programs which focus on providing nutrition to school going children (Niazi et al., 2012), micronutrient initiatives to address deficiencies such as iron, iodine, vitamin A, and zinc, among other micronutrients (Nutrition International, 2020), and a national nutrition program which targets various aspects of nutrition as well as maternal and child, infant, adolescent and adult nutrition (Ministry of National Health Services Regulation and Coordination, 2020). A specific focus on gender in nutrition is reflected in the *Tawana Pakistan Project* (Badruddin et al., 2008). This focused on increasing primary school enrollment among girls in villages, and also on combating malnutrition by providing them with freshly prepared meals is a step in the right direction. The program saw a reduction in malnutrition, stunting, and wasting in the girls in the target schools. It also saw an increase in the attendance of girls. Similarly, in other countries and municipalities such as Bangladesh and Philadelphia, nutrition interventions that have focused specifically on incorporating a gender dimension have been successful in mitigating some of the bias towards female children (Lannotti et al., 2009; Núñez et al., 2015).

While interventions targeted towards female children in the household can have positive impacts in the short to medium term, societal change will be required in the longer term to prevent discrimination against women in all forms, including nutrition deprivation (Haddad & Zeller, 1996). Such a shift involving female empowerment and expanded control of resources by women can enable greater investments in child health (Allendorf, 2007; Handa, 1996).

Second, we find evidence for an income-nutrition gradient for both *WAZ* and *WHZ* scores, suggesting that higher income, in our sample, translates into better health outcomes**.** Our findings with respect to income and *WAZ* and *WHZ* scores raise questions around how poverty alleviating interventions should be designed. In recent years, there has been an increase in cash transfers to households to improve child health outcomes. However, there is mixed evidence on their success in alleviating poverty and improving nutrition outcomes globally (Barham & Maluccio, 2009; Baulch, 2010; Gertler, 2004) and in Pakistan (Fenn et al., 2017; Jahangeer et al., 2020; Nayab & Farooq, 2014). For instance a recent study in Pakistan found that cash transfers did not lead to an improvement in anthropometrics (Jahangeer et al., 2020). However, another study found that they led to positive impact on health outcomes (Nayab & Farooq, 2014). More recent studies suggest that cash transfers coupled with behavioral interventions can lead to more promising results as they not only target income increase but also increase consumption of health inputs (Ahmed et al., 2019).

In our study, we investigated the relationship between income and dietary diversity, intake of micronutrients such as Vitamin A, and adoption of childhood immunization as potential health inputs, which may explain the positive income-child health gradient. We found that increase in income was associated with greater food diversity score, and higher likelihood of egg and meat consumption for the children. This result is consistent with previous studies indicating a positive relationship between income and dietary diversity and other health inputs (Annim & Frempong, 2018; Singh et al., 2020), which in turn result in better child health outcomes. However, gains in the consumption of these health inputs due to increase in income are higher for male children as compared to female children. This suggests that behavioral interventions which educate households on the benefits of a more equitable intrahousehold allocation, diversified diet and other health behaviors, such as seeking medical care, alongside cash transfers can help in creating an environment which may lead to better health outcomes (Adato & Bassett, 2009; Ahmed et al., 2019; Manley et al., 2013). Such interventions can be built into programs such as the EHSAAS welfare program in Pakistan which target the bottom 20% with cash transfers to raise per-capita incomes (Poverty Alleviation and Social Safety Division, 2020).

While targeted programs can have beneficial outcomes in the short to medium term, over the longer term, a greater commitment by the government for more broad based social welfare programs can enable greater and more equitable redistribution of resources (Korpi & Palme, 1998).

## Conclusion

Using Pakistan as a case, we estimated the relationship between income and child health outcomes and if higher income translates into more diversified nutritional intake among male and female children. We found that an increase in *per-capita income* is associated with an increase in WAZ and WHZ scores but not HAZ scores and the magnitude of income-nutrition gradient among boys was higher. In terms of the diversity of nutritional intake, we find that an increase in income is associated with improved dietary diversity and increased consumption of most food items. Again, gains appear to be higher among male children. Our findings point towards a need to design gender-sensitive health interventions and raise important avenues for discussion with regards to designing poverty alleviating interventions.

## Appendix

See Tables

**Table 6:** Income-nutrition gradient—Robustness checks

	Full Sample (1)	Full Sample (2)	Boys (3)	Boys (4)	Girls (5)	Girls (6)
Height-for-age regressions						
*Per Capita Income*	0.149 (0.121)	0.083 (0.144)	0.266* (0.146)	0.341** (0.166)	0.078 (0.188)	− 0.057 (0.213)
No. of Obs	856	856	445	445	411	411
Weight-for-age regressions						
*Per Capita Income*	0.378*** (0.100)	0.323*** (0.106)	0.446*** (0.147)	0.536*** (0.178)	0.333** (0.132)	0.175 (0.138)
No. of Obs	857	857	443	443	414	414
Weight-for-height regressions						
*Per Capita Income*	0.296** (0.126)	0.360** (0.142)	0.353** (0.162)	0.433** (0.193)	0.237 (0.201)	0.292 (0.213)
No. of Obs	799	799	417	417	382	382
Controls	No	Yes	No	Yes	No	Yes
District Dummies	No	No	No	No	No	No

Standard errors are clustered at the household level

Control variables include *Household Size, Age of Household Head, Child’s Gender*, *Child’s Age in Months*, *Mother’s Education, Father’s Education*

***, **, *Indicate significance at 1, 5 and 10% respectively

6 and

**Table 7:** Income-nutrition gradient

	Full Sample (1)	Boys (2)	Girls (3)
Height-for-age regressions			
*Income quartile 2*	0.149 (0.184)	0.177 (0.215)	− 0.087 (0.294)
*Income quartile 3*	0.091 (0.173)	0.192 (0.224)	− 0.152 (0.248)
*Income quartile 4*	0.048 (0.218)	0.272 (0.277)	− 0.454 (0.351)
No. of Obs	856	445	411
Weight-for-age regressions			
*Income quartile 2*	− 0.061 (0.169)	0.129 (0.237)	− 0.44** (0.216)
*Income quartile 3*	0.176 (0.165)	0.204 (0.252)	0.029 (0.205)
*Income quartile 4*	0.391** (0.185)	0.570** (0.270)	0.067 (0.235)
No. of Obs	857	443	414
Weight-for-height regressions			
*Income quartile 2*	− 0.152 (0.197)	− 0.116 (0.275)	− 0.352 (0.279)
*Income quartile 3*	0.192 (0.207)	0.211 (0.312)	0.030 (0.268)
*Income quartile 4*	0.253 (0.218)	0.418 (0.307)	0.011 (0.290)
No. of Obs	799	417	382

Standard errors are clustered at the household level

Control variables include *Household Size, Age of Household Head, Child’s Gender* (in column 1 only), *Child’s Age in Months*, *Mother’s Education, Father’s Education and District dummies*

***, **, * indicate significance at 1, 5 and 10% respectively

7.

## Footnotes

1 While the survey also includes questions on vegetable and fruit intake of children, there is a lot of missing information in those variables and therefore we decided to not use those questions.

2 Table A1 provides robustness checks with fewer controls. Our estimates remain stable and robust and we observe that gains from increase in income are greater for boys as compared to girls. Table A2 provides similar regressions with income coded in quartiles. Again, the gains seem to be larger for boys as compared to girls for both WAZ and WHZ outcomes. Furthermore, the gains seem to increase with the increase in income-levels.
